# Multicultural Adaptation of Mighty Girls for Widespread Dissemination: Pilot Study, App Development and Usability Testing, and Gauging Parent Support With Focus Groups

**DOI:** 10.2196/24937

**Published:** 2021-06-02

**Authors:** Anne E Norris, Roxana Delcampo Thalasinos, Michael L Hecht

**Affiliations:** 1 REAL Prevention, LLC Oviedo, FL United States; 2 School of Nursing and Health Studies University of Miami Coral Gables, FL United States; 3 REAL Prevention, LLC Clifton, NJ United States; 4 Department of Communication Arts and Science Penn State University State College, PA United States

**Keywords:** implementation science, mobile apps, peer influence, early intervention, adolescent health

## Abstract

**Background:**

Taking evidence-based interventions to scale is a challenge for prevention science. Mighty Girls is an evidence-based sexual health intervention program that combines classroom sessions with novel, cutting-edge technology (digital puppetry). The program was developed for 7th grade Latinas, but US school and community demographics rarely allow interventions targeting a single ethnic group. Additionally, digital puppetry is costly to scale up, and parent disapproval often prevents successful dissemination of adolescent sexual health programs. Intervening steps along the scaling-up pathway are needed to adapt the program prior to scaling up for dissemination.

**Objective:**

The aims of this study were to create a multicultural adaptation of the Mighty Girls program using a mobile app that is less costly to disseminate and is acceptable to parents of 7th grade girls.

**Methods:**

This study used a three-phase process to adapt Mighty Girls into Mighty Teens. All phases used purposive (nonprobability) sampling of low-income, multicultural, urban metropolitan groups (7th grade girls and their parents) within central Florida. Phase 1 involved two videotaped implementations of a multicultural adaptation of the classroom sessions, one involving focus groups (N=14) and the other serving as a single-group pretest-posttest pilot study (N=23). Phase 2 involved development of a narrative cell phone app prototype, which was subjected to usability testing (N=25). App usability and engagement were assessed qualitatively (observation, focus group, open-ended questions) and quantitatively. Phase 3 used focus groups to assess parent support for the program (N=6). Qualitative data were analyzed using descriptive content analysis. Quantitative data were analyzed using descriptive statistics and paired *t* tests.

**Results:**

Qualitative findings supported classroom sessions being multicultural, and identified simple changes to improve engagement and learning. Quantitative findings from the second classroom session implementation pilot study indicated a significant pre-post difference in intention to delay sexual intercourse (*P*=.04). App usability and appeal were supported by a System Usability Scale score of 76 (exceeding 68 per the industry standard) and 83% (20/24) of participants agreeing they would recommend the app to friends. Parents (mothers) expressed only positive regard for program goals, and classroom session and app activities.

**Conclusions:**

This study adapted Mighty Girls into an engaging, easier-to-disseminate, multicultural program, termed Mighty Teens, that uses a narrative-generating app to support behavior change, and is likely to be accepted by parents of 7th grade girls. This study also provides evidence of the preliminary effectiveness of Mighty Teens classroom sessions. The sampling method and sample size were appropriate for adaptation, but research involving a more representative US sample is needed to confirm multicultural fit, parent receptivity, and program effectiveness. Study implications include integrating app use throughout the classroom sessions to build narrative-generating skills across the program and increasing the number of narratives produced, which should in turn increase the program’s behavior change potency.

## Introduction

Taking evidence-based interventions to scale is a challenge for prevention science [1,2] because scaling up interventions for widespread dissemination is not always straightforward [3]. Scaling up is particularly challenging in sexual health interventions with traditional support from the literature focused on monocultural tailoring [4,5] and the focus of the US National Institutes of Health on innovation. Although these are certainly worthy goals, they are often at odds with achieving scale, particularly for school-based programs. Additionally, political and ideological issues, especially parent disapproval [6], can create barriers to dissemination [7,8]. Failure to consider these issues prior to scaling up for widespread dissemination can threaten the successful dissemination of evidence-based programs [9].

The purpose of this paper is to illustrate the steps taken to adapt Mighty Girls, an evidence-based sexual health intervention program [10], prior to scaling it up for widespread dissemination within US public school systems. Mighty Girl’s program design is guided by a theoretical framework that integrates adolescent development theory [11], social cognitive theory [12], and narrative engagement theory [13]. The first two theories define program goals: delaying initiation of intercourse by building efficacy and skills in decision-making, goal-setting, risk evaluation, and resistance to peer pressure and media messages implying teen sex as a common behavior. By contrast, the narrative engagement theory defines the overarching program strategy: using narrative-generating activities to (a) build program skills, and (b) combine and imbed skill knowledge and program messages in memory. The resulting Mighty Girls program includes 6 classroom sessions and a computer game, DRAMA-RAMA, that generates a first-person narrative about responding to peer pressure in a simulated early adolescent world (Textbox 1).

**Mighty Girls program components and corresponding components in the Mighty Teens adaptation.**

Sessions began with “Mighty Moments,” a kinesthetic learning experience of relationally competent resistance communication skills developed by the second author. Session objectives are listed with the original (Mighty Girls) program session title. Objectives were retained in the adaptation with minor modifications.

**Choices & Results (split into two sessions in Mighty Teens: Goals and Choices & Results)**

- Identify personal goals (modified as “Goals” in Mighty Teens)
- Understand the results of everyday choices (modified as “Choices & Results” in Mighty Teens)
- Relate choices and results to goals (modified as “Choices & Results” in Mighty Teens)

**What’s Risky? (maintained as “What’s Risky?” in Mighty Teens)**

- Explain why some behaviors could be considered risky
- Identify potential results of choosing to engage in risky behaviors
- Discuss what increases or decreases the risk of a specific behavior
- Identify what behaviors are risky for oneself

**The Avoid Skill (modified as “Avoid & Leave” in Mighty Teens)**

- Define Avoid Skill
- List 3 methods for avoiding an uncomfortable or risky situation
- Avoid the Mighty Girls way: being considerate, confident, and convincing

**The Refuse Skill (modified as “Refuse & Explain” in Mighty Teens)**

- Explain differences between aggressive, passive, and assertive communication
- Demonstrate matching voice and body language using Refuse Skill
- Refuse the Mighty Girl way: being considerate, confident, and convincing

**Media Influences (modified as “Challenging Media Messages” in Mighty Teens)**

- Explain purpose of media
- Think critically about advertisements and TV show messages
- Identify positive and negative messages about girls and women in the media
- Identify ways in which teens on popular TV shows are different from and similar to teens in real life

**Wrap-up & Review**

- Model results–based choices
- Demonstrate Mighty communication: using considerate, confident, and convincing words and body language.

**Tech Component to Support and Sustain Behavior Change (modified as a cell phone app in Mighty Teens)**

- Digital puppetry computer game, DRAMA-RAMA

The Avoid Skill and The Refuse Skill sessions were adapted from “keepin’ it REAL” [14]. Other sessions were cocreated by the first and second authors.

Mighty Girls has three features, which the nonadoption, abandonment, scale-up, spread, and sustainability (NASSS) framework identifies as likely to result in failure to scale up and disseminate [9]. First, the program was developed for Latinas. This monocultural focus is at odds with successful dissemination within US public school systems given US school and community demographics [15]. Additionally, research findings argue for greater effectiveness of multicultural, relative to monocultural, program interventions in general [16] and within US school settings in particular [17,18]. Second, DRAMA-RAMA relies on human-in-the-loop technology (digital puppetry) [10]. Advances in artificial intelligence are not yet sufficient to reduce this cost (current personnel and equipment costs estimated at US $20,000 per school), making the intervention not sustainable within US public school systems. An alternative, less costly narrative-generating technological component such as a smartphone app would address this sustainability barrier. Third, it is wise to gauge potential parent support for any school-based sexual health program [6], particularly one that uses a smartphone app [19], early in the scaling-up process when changes are easier to accommodate.

## Methods

### Overview

**Figure 1:**
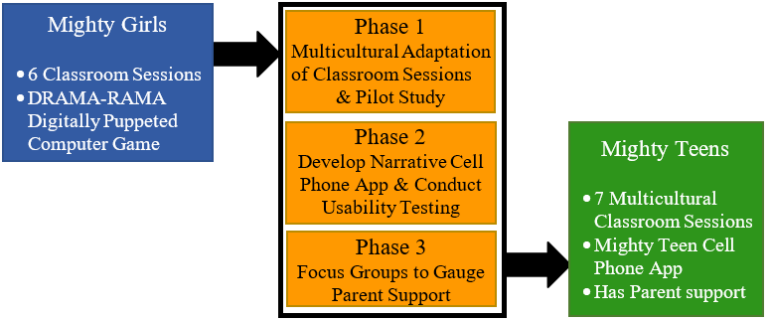
Adapting Mighty Girls into Mighty Teens, a more scalable and easier to disseminate program.

All three phases of program adaptation (Figure 1) used purposive [20] (nonprobability) sampling of groups living in a low-income, multicultural, urban metropolitan area within central Florida. Recruitment and study procedures were approved by the Prevention Strategies LLC Institutional Review Board and participating school district. Below, we present information specific to each phase, one phase at a time.

### Phase 1: Multicultural Adaptation of Classroom Sessions and Pilot Study

#### Design

Phase 1 used an iterative process involving consultation and two implementations of the classroom sessions, with the second implementation serving as a pilot study. After each iteration, the classroom sessions were revised.

Three changes to program materials were made in preparation for phase 1. First, session slide sets were revised to ensure representation of a variety of cultural/ethnic groups. Second, content on narrative writing was added to the final “Wrap-up & Review” session. Third, ordering of the Avoid Skill and Refuse Skill sessions was reversed relative to their positioning in the original program. This last change was driven by insights from implementing the original program [10]. Both sessions were adapted from an efficacious, early-adolescent substance use prevention program, “keepin’ it REAL” [14], which stands for the relationally competent resistance communication skills Refuse, Explain, Avoid, and Leave. Only Refuse and Avoid were taught in Mighty Girls because formative work indicated that Explain could elicit more peer pressure by inviting peers to argue [21], and Leave could not be used in DRAMA-RAMA.

#### Consultation

Consultants included a female middle school staff member and three adult women in their early 20s (two African American women and one biracial African American/Native American woman). The latter were recruited as research assistants through Indeed, a worldwide employment website for job listings. These young women all lived in the same low-income, multicultural, urban metropolitan area in which the study was being performed. We specifically empowered them by emphasizing during recruitment and task directions that they brought a unique and valuable expertise critical to the success of the research effort (this message was repeated throughout phase 1 and phase 2 for all tasks involving these women). They were tasked with reviewing the Mighty Girls classroom session program manual and slide sets for language, terminology, and image inclusivity (ie, not heterosexist or specific to a particular ethnic group) and making recommendations.

#### Participants, Procedures, and Measures

Participants in both implementations were girls enrolled in the 7th grade (aged 11-14 years) at two multiethnic middle schools in an urban metropolitan area within central Florida. No participant had difficulty speaking, reading, or writing in English, and on average used only English, or English more than other languages, for these activities (ie, high level of acculturation). Participants of implementation 1 were recruited at a low-income (67% reduced/free lunch, in accordance with criteria to be designated as a “Title I” school: 65% of students/families qualify directly for supplemental nutrition assistance program, homeless, migrant, Medicaid, or foster care as verified by the state) middle school with 80% minority enrollment. Two girls dropped out after session 4 due to repeated disruptive behavioral issues. Participants of implementation 2 were recruited at a second low-income (52% reduced/free lunch) school with 69% minority enrollment (top 5% of state schools for enrollment diversity). Participants at both schools were recruited with: (1) flyers posted at the school; (2) a message sent out via phone by the principal to parents of 7th grade girls; and (3) the three research assistants discussing the study and handing out study packets during 7th grade lunch (packets contained the principal’s letter of support, parental consent forms, and screening form assessing parents’ culture/ethnicity and child’s English language fluency).

Each implementation involved a two-person multicultural teaching team consisting of either the first or third author in the teaching role and one of the three research assistants in the teaching assistant role (ie, Hispanic or non-Hispanic White teacher paired with African American or multiethnic [African American/Native American] teaching assistant). This team also acted as participant observers who made postsession notes about the session they had taught, identifying what worked and did not work, and providing feedback on the cultural relevance of the wording and examples used to explain concepts.

Teaching assistant role training involved completing session activities in the role of a participant, and provided an opportunity to try out a training approach that combined two 2-hour face-to-face sessions with a “homework” assignment similar to what might be used for training when the program is taken to scale. All three research assistants spontaneously reported how much they liked their training, particularly the “homework” (using communication techniques with friends and family).

Both implementations were videotaped. The first implementation involved postsession focus groups, and the second was a one-group, pretest-posttest pilot study (not powered for significance).

#### First Implementation

Participants at the first school completed a brief demographic questionnaire immediately prior to the first session that assessed age; birth country; race and ethnicity; acculturation (use of first language relative to English for those who spoke more than one language) [22,23]; and whether lunch was free, reduced in price, or full price.

Each Mighty Teens classroom session was delivered and then followed by a short focus group led by the teaching team. Focus group questions were displayed on slides. Participants wrote responses on large pieces of paper posted on walls or on anonymous index cards, depending on the question. They then voted (ie, agreed/disagreed) on themes identified in comments written publicly and anonymously, and responded to probes for more details or new thoughts emerging in response to other teens’ comments.

#### Second Implementation/Pilot Study

In lieu of focus groups, participants at the second school completed a paper-and-pencil pretest immediately prior to the first session (also containing the same demographic items used in the first implementation) and completed a posttest immediately after the last session. All measures had been pretested or used with this age group previously [10,24].

The pretest and posttest contained a short self-efficacy scale, along with items assessing resistance and sexual intentions, and intention to postpone sexual intercourse. The 9-item self- efficacy scale comprised 5 items from the 12-item measure reported by DiIorio et al [25] (eg, say “no” to sex even if the other person says they will break up with you if you don’t have sex) and 4 items created by the first author to assess self-efficacy regarding specific program resistance behaviors [10]. These 4 items were: (1) stop someone who is pressuring you to do sexual things without making them angry, (2) leave a party or dance club where sexual things are going on without being made fun of, (3) avoid situations where you know other people will be making out, and (4) say no in a serious way and stick to it when someone you like is pressuring you to do sexual things. Response options range from 1 to 4 with labels for endpoints and midpoints (1=not at all sure; 3=moderately sure; 5=completely sure). Higher scores indicate greater resistance self-efficacy. Cronbach α was .84.

All intention items used the same response options: definitely not (1), probably not (2), probably yes (3), and definitely yes (4). Higher scores indicate greater intention. Psychometric analysis argued for treating intention types as measures of separate constructs, not part of a single multi-item intentions scale. Resistance intentions were assessed by a question related to a particular response strategy when asked “to do something you do not want to do.” Response strategies included: not resisting (go along with what they want me to do), resisting by using relationally competent communication strategies (4 items, including suggest doing something else; α=.62), or resisting by using a nonrelationally competent strategy (tell them I don’t want to because it’s stupid). Kirby et al’s [26] 3-item sexual intention measure was used to assess willingness to engage in sexual intercourse (eg, I would have sex now to keep someone I cared about romantically as a boyfriend/girlfriend); Cronbach α was .79. Intention to postpone sexual intercourse was assessed with a single item: I want to wait to have sex until I am older.

#### Data Analysis

Demographic responses were summarized with descriptive statistics. A paired *t* test was used to assess pretest-posttest differences in self-efficacy, resistance and sexual intentions, and intention to postpone sexual intercourse (second implementation only).

The first and second authors reviewed classroom session videos (both implementations) for signs of engagement, restlessness, and confusion. Engagement was defined as girls raising their hands to participate, smiling, looking at the teaching team or slide presentation when not engaged in a task, working on tasks, or easily redirected from chatting with peer(s) back to the main discussion and remaining attentive. Restlessness was defined as fidgeting, repeatedly talking with peer(s) and not easily redirected back to the task, complaining about the activity, being bored, or not having fun. Confusion was defined as a facial expression in which the nose and forehead were scrunched up in a type of frown, or complaints about being confused or not understanding task directions.

The first author also reviewed and performed a descriptive content analysis [27] of the teaching team postsession notes (both implementations) and postsession focus group videos (implementation 1 only). Content (eg, sentence or section of notes; participant response or portion of response if expressing multiple ideas) was sorted into one of three categories: (1) liking or positive, (2) disliking or negative, and (3) confusing or not clear. Improvement suggestions noticed in this process were also marked. Next, information within categories and improvement suggestions were each reviewed for themes and multiple instances, summarized, and then discussed with the study team.

### Phase 2: Development of a Narrative Cell Phone App and Usability Testing

#### Design

Phase 2 began with app development that culminated in usability testing. The app prototype was initially developed for Android phones given Pew Foundation reports [28] indicating greater Android use by low-income families living in the urban metropolitan area within central Florida in which this research was being performed. However, the next iteration of the app will be built for both Android and iPhone cell phones.

Two theories guided app development. First, the theory of fun [29] stresses the importance of engagement for app uptake, and defines engagement as the result of positive affect, fun, low predictability, novelty, and the right amount of challenge (not too little or too much) experienced while interacting with the app. Second, narrative engagement theory [13] stresses the centrality of engagement to behavior change. According to this theory, engagement mediates overall effectiveness of narrative creation, a phenomenon that builds self-efficacy through cognitive rehearsal [12] while simultaneously imbedding intervention messages (eg, concepts) in a narrative [13]. App use was planned for nonschool settings (eg, home, public library), necessitating a simple and intuitive interface. Additionally, the interface needed to guide users in creating their own “Mighty Teens” electronic story of resistance (similar to the events that occurred with DRAMA-RAMA in the Mighty Girls program). Stories were to be recorded, mirroring natural storytelling.

**Figure 2:**
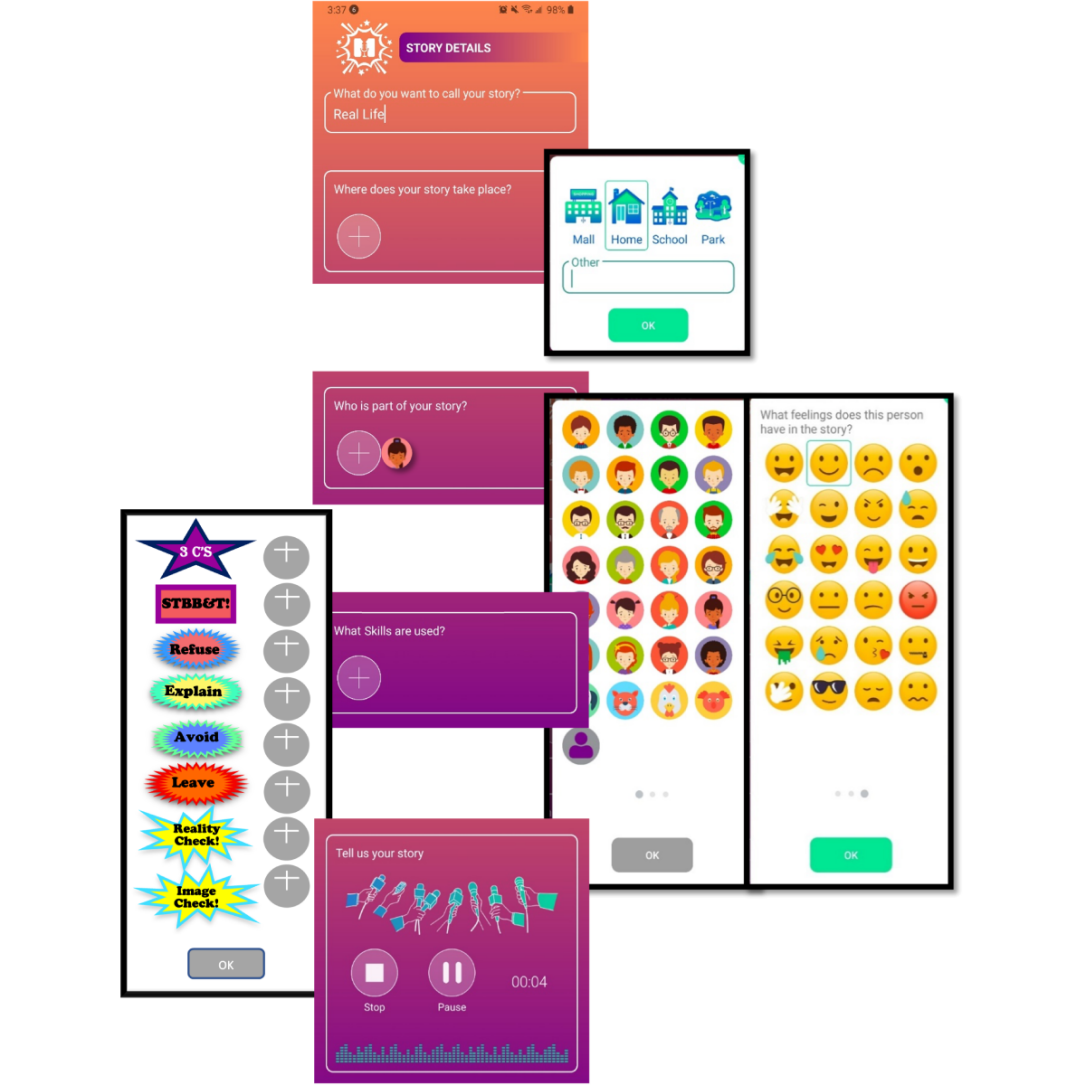
Mighty Teens app prototype story-creating screen with pop-ups allowing selection of places, characters with as many emotions (represented as emoticons) as needed, and Mighty Teens skills.

Both the app and a secure, password-protected dashboard (backend accessible to administration staff) were developed using a collaborative approach, involving the authors and Margaret Broucek, Principal of Tapp.technology who was a phase 2 technology partner (Textbox 2). Tapp.technology iteratively created screen designs in response to feedback from the authors and the three research assistants, arriving at the designs used to create the prototype for usability testing (Figure 2).

**Required prototype features and functionalities.**

**Mighty Teens app**

- Intuitive interface with minimal text or direction
- Record oral story. Text input only required for naming story
- Present users with icons representing places, characters and their emotions, and Mighty Teens skills from which to select
- Provide users with button to click to access definitions of individual Mighty Teens skills
- Provide users with a button to click to record story while viewing selected icon places, people and their emotions, and Mighty Teens skills
- Allow users to listen to their story, and submit, rerecord, or delete it
- Send user stories using secure encrypted transmission to Mighty Teens dashboard

**Mighty Teens Dashboard**

- Secure, password-protected
- Allow direct entry and edit of information into sortable fields with controlled access to such privileges
- Usernames grouped by implementer and implementation school/clinic site
- Implementation school/clinic site with street, address, state, and zip code information, and grouped by organization
- Store usernames, app access codes, addresses, and story submissions
- Assign various levels of access to all stored information

#### Participants and Procedures

Phase 1 participants were invited to participate in usability testing as part of their study participation. However, some were unable to attend due to transportation issues or schedule conflicts (eg, needed for sport team practice), resulting in a sample of 25 participants. Almost all participants (92%, 23/25) created and recorded at least one story. Two participants interacted with the app but did not record (or deleted recordings of) their stories. One of these participants did not want anyone to listen to her story. It is unclear if the other had difficulty with the recording feature or felt similarly. Although 25 girls participated in app testing, only 24 completed written assessments of usability and engagement.

Usability testing was performed at each school involved in phase 1 with hot spots, approximately 1 month apart. Participants accessed the app using either their own Android cell phone or a “testing” cell phone provided by the research team.

When participants arrived at their respective school, they were split into two groups with each group assigned to a different classroom. Once in their classroom, participants were split into dyads and a research team member was assigned to observe each dyad. Those that finished early were free to record additional stories. Research team members took ethnographic field notes describing participants’ verbal and nonverbal behaviors during app use, but were unable to see the participants’ screens unless shown to them. Research team members debriefed immediately after testing. Notes from this meeting became part of the ethnographic field notes.

After using the app, the girls completed written assessments of usability and engagement. Following this, a brief focus group discussion was held with the first or second author in the role of facilitator and a member of the research team in the role of cofacilitator/notetaker.

#### Measures

##### Quantitative Measures

Self-reported usability was assessed with an adaptation of the System Usability Scale [30] in which the word “system” was replaced with “app,” and wordiness and reading level were reduced to make items more accessible to teens. For example, the original item “I think that I would like to use this system frequently” was revised to “I would use this app a lot.” Response options (strongly disagree, disagree, neutral, agree, strongly agree) were unchanged.

Consistent with the original scoring process [30], responses were assigned a value of 0 (strongly disagree) to 4 (strongly agree) with even-numbered items reverse-scored, and scores were summed with the result multiplied by 2.5 to create a usability score ranging from 0 to 100. Cronbach α for the adapted measure was .80. Validity was supported by correlations with the app being interesting (*r*=0.60, *P*=.002), task difficulty (*r*=–0.44, *P*=.048), and flow (*r*=0.45, *P*=.03).

Usability was also assessed with an open-ended question asking what could be done to improve the app, and task difficulty was assessed with a closed-ended question: “It was hard to think of an idea to use for the Mighty Teens app.” Response options were strongly disagree (1), disagree (2), agree (3), and strongly agree (4).

Self-reported engagement was measured with 6 items adapted from those used in DRAMA-RAMA development by replacing the word “game” with “app” and referring to writing stories instead of interacting with game characters [10]. A single item assessed whether the app was fun to use. A 5-item Likert scale consisted of the following items: three items describing the app experience (fun way to create a story, interesting, boring [recoded]), one item asking about using the app for a school assignment, and one item asking about recommending the app to friends. Response options ranged from strongly disagree (0) to strongly agree (3), without a neutral midpoint.

##### Qualitative Measures

Ethnographic field notes were used to assess usability and engagement presence and issues. Research team members were trained to collect these notes and used a checklist as a guide to verify that they had commented on all assessment domains. The checklist prompted assessment of (a) verbal and nonverbal signs of positive and negative affect, and (b) problems with usability indicated by participants having completed the storytelling task (eg, long delays, facial expressions of frustration, asking questions).

Focus group interviews consisted of open-ended questions that asked for general impressions, likes and dislikes, and recommendations for improvement. These questions were followed by requests for feedback on specific screens or parts of screens.

#### Data Analysis

Ethnographic field notes, focus group notes, and participant responses to the open-ended usability question were analyzed using descriptive content analysis [27], similar to phase 1. However, the first author sorted units into categories, and then identified and presented a summary of themes represented by these categories to the usability testing team, along with copies of the raw qualitative data. The summary was finalized in the resulting discussion.

Participant stories were coded for narrative structure and reference to Mighty Teens program skills using two dichotomous (0, 1) variables. Narrative structure was defined as three complete components (1). A reference to a Mighty Teens program skill (1) required the participant to describe a character correctly using (or teaching another character to use) at least one skill. It was not necessary to use the exact program name for the skill. However, not referencing a skill or naming one without any application were both coded as no reference (0). Interrater agreement was 100%.

### Phase 3: Focus Groups to Gauge Parent Support 

#### Participants, Procedures, and Measures

Mothers and fathers of 7th grade girls involved in the second implementation were invited to participate in a focus group (a) by flyers included in the study packets given out to their daughters, and (b) during the parental consent process for their daughters’ participation. No fathers expressed interest in participating or were involved in the consenting process.

Scheduling of focus group participation coincided with the first national lockdown in response to the COVID-19 pandemic. Parents were distracted, under distress, with other activities competing for their attention, complicating consenting and scheduling (eg, parents who had previously expressed interest did not return phone calls). Scheduling and COVID-19 accommodations resulted in two focus groups involving 6 mothers as participants.

Focus groups were conducted using Zoom conference technology, which was challenging because the participants seemed unfamiliar with Zoom and video conferences in general. However, the facilitator (second author) was able to assist by phone, and familiarize participants with the process and importance of confidentiality and privacy once they opened Zoom.

Focus group interviews consisted of open-ended questions about (1) what the parents had heard about Mighty Teen classroom sessions or the app from their daughters, and (2) the parents’ feelings about and challenges related to their daughters’ use of apps and cell phones. Information about classroom session topics and activities, and about the app was provided once parents shared what they had heard. Feedback was then solicited, followed by specific probing related to their daughters’ use of the app, her doing so privately, and supervision preferences and concerns.

#### Data Analysis

Descriptive statistics are used to summarize the mothers’ ethnicities. Focus group recordings were transcribed verbatim and analyzed using descriptive content analysis [27], similar to phases 1 and 2. However, the first and second authors independently sorted units into positive/negative categories and summarized themes within the categories. No differences in categorization or meaning of theme labels were noted.

## Results

### Participant Characteristics

Participant demographic characteristics for all three study phases are provided in Table 1.

**Table 1:** Demographic characteristics of participants by study phase.

Characteristic		Phase 1: Implementation				Phase 2: Usability testing (N=25)			Phase 3: Parent focus groups (N=6)		
		1 (n=14)		2 (n=23)							
Age (years), median (range)		13 (12-14)		13 (11-14)	13 (11-14)			—^a^			
Qualifies for reduced/free lunch, n (%)		11 (85)		16 (70)	19 (76)			N/A^b^			
**Cultural/ethnic group, n (%)**											
	African American	5 (36)		5 (22)	8 (32)			_2 (33)_c			
	Arab American	0 (0)		1 (4)	1 (4)			0 (0)			
	Bahamian/Black Caribbean	2 (14)		2 (9)	2 (8)			_1 (17)_c			
	Haitian	0 (0)		4 (17)	3 (12)			_1 (17)_c			
	Hispanic/Latino	4 (29)		5 (22)	5 (20)			_1 (17)_d			
	Indo-American	1 (7)		1 (4)	1 (4)			0 (0)			
	Non-Hispanic White	1 (7)		2 (9)	2 (8)			_1 (17)_d			
	Multicultural (African American and Haitian; African American and Hispanic/Latino; Navajo and Non-Hispanic White; Bahamian and Haitian)	1 (7)		3 (13)	3 (12)			0 (0)			
US (mainland)-born, n (%)			11 (79)	20 (87)			21 (85%)	—			
High level of acculturation, n (%)			12 (86)	20 (87)			22 (88%)	—			

^a^Data not collected.

^b^N/A: not applicable.

^c^Part of same focus group.

^d^Part of same focus group.

### Phase 1: Multicultural Adaptation of Classroom Sessions

#### Consultation

The consultants recommended taking more time in session 1 to explain the program, and making two changes to increase program language inclusivity: (1) replace “girls” with “teens” (including in the program title); and (2) use nongender-specific names and pronouns to avoid gender identity and sexual orientation biases. These changes were recommended to make teens feel included regardless of gender identity or sexual orientation. The only exception was in the Media Influences session, where the focus on how girls and women are depicted in advertisements was viewed as valuable. The three young adult consultants also reported (without prompting) how much they liked the program and wished it had been offered to them when they were in middle school, lending support for its multicultural potential.

#### Implementations

##### Overview

Review of classroom session videos and teaching impression notes from both implementations failed to identify instances indicating a lack of multiculturalism in terminology or directions, including those related to resisting peer pressure. For example, expressions of confusion tended to be made by multiple teens who were not more likely to be of a particular cultural/ethnic group than other participants. Moreover, the teaching teams of implementations 1 and 2 failed to report any instances of program content, directions, or examples feeling “off,” “needing to be translated,” and similar. Below, we discuss the implementation-specific findings.

##### First Implementation

Analyses of focus group and classroom session videos, and teaching impression notes identified two pedagogical issues and one opportunity to fine-tune the content. First, participants appeared to be confused when identifying goals in session 1, indicating that more time was needed for teaching this content, resulting in the decision to split this session in two, with the first objective retained in the new Goals session and the remaining two objectives addressed in the new Choices & Results session (Textbox 1). Second, fidgeting and restlessness were noted during the session 3 content when the resistant communication skill Refuse was presented, indicating a need for physically active learning (eg, having the class stand and share in acting out the Refuse skill). Aside from these issues, all other disengagements could be linked to one or two participants with a preexisting peer conflict documented in teaching impression notes. Third, review of Refuse and Avoid session videos led to a discussion with the teaching team that resulted in (a) adding a clear definition of Explain, but restricting it to a type of vague explanation that is difficult for peers to argue with (eg, “I’m not into that”); and (b) defining Leave as a response that can be combined with Refuse, Explain, or Avoid. In the original program, this content has been presented more as a type of Refuse or Avoid skill but with little emphasis. As a result of this change, session objectives were slightly modified to include defining and applying Explain and Leave, respectively, and session titles were modified to “Refuse & Explain” and “Avoid & Leave.”

##### Second Implementation and Pilot Study

The teaching team reported that splitting session 1 into two different sessions increased class discussion time and allowed for the goal content to be covered more slowly, improving participants’ grasp of this content. Review of videos indicated that any confusion initially present declined over the period in which the goal content was presented. Similarly, fidgeting and restlessness during the Refuse skill content presentation appeared to be eliminated by increasing the physical learning of this skill. Nevertheless, the teaching team reported that, overall, it was difficult to deliver all content within 45 minutes and still have sufficient time for discussion. This was particularly true for sessions 4 and 5, which had been revised to include content on Explain and Leave, respectively, and also for session 7 to which content on narrative writing had been added. In other words, all three sessions need to be revisited to streamline/reduce their content.

Engagement was evident across all sessions. Any disengagement could again be linked to a preexisting peer conflict noted in the teaching impression notes.

Quantitative analyses identified a significant pre-post increase in intention to delay sexual intercourse (*t*_14_=–2.26, *P*=.04). No significant pre-post increases in resistance self-efficacy (*P*=.71) or resistance intentions (*P≥*.30), or decreases in sexual intentions (*P≥*.17) were observed.

### Phase 2: Narrative Cell Phone App Usability Testing

#### Qualitative and Quantitative Measures

We could not detect any substantive differences between the results obtained for the two usability testing sessions. Hence, results presented herein are combined across sessions. The observed time to complete a task ranged from 6.30 to 13 minutes (median 6.67). The prototype did not track the time spent between opening the app and submitting a story, and teens were offered the opportunity to record their stories outside if they did not want to be overheard. Hence, the observed time to complete a task may be confounded with the time spent visiting with friends, a behavior observed at the same time the recording was presumed to be occurring, arguing for it being a “high-end” estimate of the actual time required.

Only 24% (6/25) of the participants created a story with a beginning, middle, and end, and less than half of the participants (44%, 11/25) created a story in which Mighty Teens skills were used. The stories reflected both same and opposite sex orientations, consistent with the tailoring expected in self-generated narratives.

#### Usability

The System Usability Scale score was 76 despite 55% (12/22) of the participants agreeing that it was hard to think of an idea to use for the Mighty Teens app. Content analysis of open-ended question responses and focus group notes indicated that about half of the participants wanted the app to have a tutorial, more directions, or prompts, whereas about half felt that it was “simple,” “easy,” or “very straightforward.”

Analysis of ethnographic field notes described some participants as frowning and stopping, as if concentrating, and others clearly stuck at the initial step of identifying a title until told they could use “My Story” as the title if they wanted to. Regardless, these participants quickly transitioned to a rush of tapping and swiping with brighter affect, and focus group notes indicated that all but one participant (disgruntled by a preexisting peer conflict) were pleased with their experience. Nevertheless, many did not like how their recorded voice sounded.

Slightly more than a third (9/24, 38%) of the participants stated in their written responses to the usability open-ended question that there was nothing needed to improve the app (eg, “no,” “nope,” “nothing”). Less than half (11/24, 46%) made one or more comments concerning actual or desired app features, and 17% (4/24) made no comments.

Descriptive content analysis of responses to the usability open-ended question ethnographic field notes or focus group notes identified ways to improve usability (Textbox 3). Triangulation of these data sources indicated that a majority of participants (80%, 20/25) were frustrated or disappointed that they could not type their story. In contrast, 20% (5/25) expressed joy and astonishment that they could record (they had expected text entry) and had quickly begun using the app with gusto. However, this latter group agreed in the focus groups that text entry would be useful if a teen was concerned about being overheard, or was mute or stuttered.

Four unsubmitted stories were found in the testing phones after the session supported participants’ suggestion to add confirmation of a successful story submission. Additionally, the focus group discussion helped clarify other usability improvements. For example, all participants wanted the character set to be more representative but some also felt that the numbers of icons in the current character set was overwhelming, arguing for customizing over expanding the existing set.

Similarly, the importance of adding an “other” category for character emotions was emphasized by comments indicating that participants considered qualities such as courageous, shy, and determined to be emotions. In contrast, comments regarding places suggested adding both icons (eg, corner store, fast food place, “jumping place” [a place for teen parties with trampolines]) and an “other” option to accommodate places that might be more regional or seasonal (eg, pool, beach).

**App improvement categories and themes identified in descriptive content analysis.**

**Usability improvement categories and themes**

- Allow both recording and text entry of stories.
- Label emoticons (cannot tell what some of the faces mean).
- Improve ability to select story elements:
  Allow multiple selections without exiting and reentering screen.
  Increase differentiation between skill versus skill definition selection (add a question mark for definition).
- Improve ability to delete characters.
- Make “controls” (pause, record, submit) on recording screen clear.
- Confirm story submission, so you know it worked.
- Increase choices and/or allow more customization:
  Current character set not sufficiently representative (nobody looked like me).
  Add selection of various different intensities of particular emotions (eg, a little angry, extremely angry).
  Add more places.
  Add “other” option for emotions and places similar to what is available for characters.
- Change to blue and red colors if app to be used by both boys and girls at some point; current colors are “girly.”

**Engagement improvement categories and themes**

- Make possible to anonymously share and chat about stories to help other teens or obtain feedback on how story creator responded (or could have responded) to a particular situation.
- Increase the fun:
  Add emoticons floating across login page.
  Have app “read back” in a male or female voice, according to user’s choice.
  Animate stories (have characters act out story).

#### Engagement

Descriptive content analysis indicated smiling, intense focus, and constant interaction with the app for 23 out of 25 (92%) participants with these same participants asking if they could use the app to make multiple stories. At least 4 out of 23 (17%) participants who requested this option did so in the time available with one participant creating 4 stories.

The word “fun” was used multiple times in all focus groups to describe the app; 83% (20/24) of participants reported they would recommend the app to their friends. Mean Fun and Like scores were 3.0 (SD 0.75) and 3.2 (SD 0.60), respectively, indicating that the participants agreed that the was app fun and liked using it. Additionally, both ethnographic and focus group notes indicated that participants found selecting icons for characters, character emotions, and places very appealing. Selecting emotions was stated as a “favorite part.”

Analysis of open-ended question and focus group data identified changes that could increase engagement (Textbox 3). One such change (sharing and chatting about stories with other teens) appeared part of a larger theme of wanting the app to have a purpose; otherwise, “why should we use it?”

### Phase 3: Focus Groups Gauging Parent Support

Descriptive content analysis of focus group transcripts indicated no obvious group differences, and no ethnic or racial differences. Hence, results for each group are combined with participants referred to as mothers because no fathers participated (Table 2). There were no negative comments about any aspect of the program.

**Table 2:** Themes identified in parent focus groups.

Theme	Exemplar quote^a^
Mothers liked program goals and content	“…she [referring to daughter] was just saying, ‘You got to be careful when you’re on social media, because you don’t know who you’re talk to who they are.’…I was Okay, she must have mentioned it because of the session…now they’re having a session and all of a sudden, they know what to do so, that I did appreciate.”
Mothers liked the Mighty Teens app	“What I find different about yours versus the others is that she can either make up a story or use her own story…The other thing that I find that your app is interesting about is the fact that it brings in emotions, which I don’t think the others do that. I like the emotional aspect of your app.”
Mothers want Mighty Teens app use monitored	“Sometimes they’re not willing to talk about it themselves,…if they shared something like that in a story, then it may put up a red flag: ‘Hey, this person needs help.’”
Sharing is a benefit to daughter and other teens	“I think if it’s in a controlled environment, as far as your company or whomever, I think that will be something that is great for the kids to share whether it’s a fictitious or whether it’s a true story…to kind of make awareness to other children…”

^a^Each exemplar quote is from a different focus group participant. Participant race or ethnicity is not reported to protect confidentiality.

## Discussion

Scaling up culturally tailored sexual health interventions can be challenging and not always straightforward [3]. This study addressed three barriers that the NASSS framework [9] argued would impede successfully scaling up and disseminating the Mighty Girls program: lack of multiculturalism, cost, and parent receptivity. Phase 1 used an iterative process involving consultation, focus groups, and pretest-posttest evaluation to produce a multicultural adaptation of the classroom sessions and a new program name, Mighty Teens. The iterative process increased program language inclusivity with respect to gender identity, sexual orientation, and culture/ethnicity, consistent with best practices for creating multicultural programs [16]. Phase 1 also provided preliminary support for efficacy of the multicultural Mighty Teens classroom sessions.

Phase 2 used a combination of qualitative and quantitative methods to evaluate app usability and engagement potential. The usability score (76) exceeded the minimum industry standard (68) for minimum or average usability [31]. Additionally, the app enabled users to self-tailor their narratives to the level of sexual orientation, which was not possible using the original program’s technical component. The app is less costly to disseminate than the Mighty Girls DRAMA-RAMA game, and potentially more powerful, because it affords a greater level of tailoring [13].

Finally, phase 3 parent focus groups demonstrated not only parent receptivity but also positive regard for the Mighty Teens program. This argues for the program’s ability to surmount the political and ideological issues surrounding sex education that typically impede sexual health program dissemination and uptake [6-8].

Looking across the findings from all three phases of this study, two themes stand out. First, the study findings suggest that Mighty Teens has high engagement potential. This argues for program success and impact because engagement predicts program participation [32], learning [33], and behavior change [13]. Second, both mothers and daughters liked and valued a narrative-generating app that allows teens to (a) select emotions for characters in a story and (b) share their stories with peers in a monitored environment. Although it is unclear if fathers would feel similarly, mother-daughter agreement suggests parents would support their daughters’ app use in the home environment. This conclusion is also consistent with the technology acceptance model’s premise that user value for particular app functionalities is critical to app acceptance and uptake [34].

This study did have limitations. Participants were purposively selected to represent various cultural and ethnic groups living within a low-income, urban metropolitan area within central Florida, and sample sizes were small, preventing us from statistical evaluation of possible cultural or ethnic differences. All parent focus group participants had previously consented to their daughters’ participation in a sexual health program, and may have been more likely to approve of sex education in general, as well as this program. Further, only mothers participated in the parent focus groups. However, research shows that fathers often defer to mothers when it comes to their daughters’ sexual health [35], and our sampling methods were suitable for adaptation purposes. Nevertheless, more cultural/ethnic differences might emerge in larger samples. Hence, we will evaluate both multicultural fit and parent receptivity in a future efficacy trial involving a more representative US sample.

Despite these limitations, this study illustrates how program developers can adapt a program prior to scaling up for dissemination so that clear barriers to implementation by targeted user organizations (ie, public schools) are addressed. Our three-phase approach models the use of specific guidelines and best practices for creating cultural [36] and multicultural adaptations [16] of evidence-based preventive interventions. For example, we involved a multicultural team of consultants (school counselor and three young adult women) familiar with and representative of a multicultural community. Our three young adult women were all intimately involved with the process of adapting the intervention in phases 1 and 2 in their roles as research assistants, and were empowered to shape this process. Our pilot study, usability testing, and parent focus groups provided data regarding intervention acceptability and efficacy as well as feasibility. Moreover, the empowered role of our research assistants illustrates how to extend the principle of coproduction of public health interventions from intervention development to adaptation of an existing evidence-based intervention [37].

Additionally, the study findings provide clear directions for three app-related program improvements. First, there are clearly two different teen subgroups that need to be accommodated: those that prefer to record a story and those that prefer to use text. Second, story creation can be a difficult task for this age group. More support for structuring stories, including a prompt to use a Mighty Teens skill, needs to be designed into the app. Our findings suggest that either narrative writing is not consistently taught in 7th grade, despite being part of the US 7th grade common core standards [38], or, if it is taught, the content and related skills can be challenging for early adolescents to master. The ability of a sexual health program to support learning of an academic skill should increase the program’s appeal to parents and public schools, thereby facilitating dissemination [6]. Third, the app should be introduced earlier and used to build narrative-generating skills over the course of the program, opening up possibilities for classroom activities to move into the app, and creating more time for class discussion. This change also enhances relevance and synergy between the app and classroom session activities, and increases the number of narratives participants create. Meanwhile, increasing the number of narratives increases Mighty Teens behavior change potency [13].

In conclusion, this paper describes a three-phase process for adapting an evidence-based monocultural sexual health program to increase the ease and success of scaling up and dissemination. Specifically, this process (a) created a multicultural version of the classroom sessions (phase 1); (b) replaced a costly technological component with a less costly easier-to- disseminate narrative-generating cell phone app (phase 2); and (c) obtained preliminary parent support for the adaptation, including their child’s use of the program’s app component (phase 3). Findings generated by this process provide preliminary support for Mighty Teens program efficacy, and insights for increasing program engagement, potency, and perceived value.

## Abbreviations

NASSS: nonadoption, abandonment, scale-up, spread, and sustainability
